# Scale-Constrained Synthetic Construction for Small-Sample Satellite Power Tower Damage Assessment Under Cross-Scale Mismatch

**DOI:** 10.3390/s26103241

**Published:** 2026-05-20

**Authors:** Yulong Liu, Qi Wen, Jianghong Zhao, Runyu Ma, Atta-ur Rahman, Xiaolin Tian

**Affiliations:** 1 School of Geomatics and Urban Spatial Information, Beijing University of Civil Engineering and Architecture, Beijing 102627, China; 2108160323015@stu.bucea.edu.cn; 2 Technology and Engineering Center for Space Utilization, Chinese Academy of Sciences, Beijing 100094, China; marunyu@csu.ac.cn; 3 Department of Geography and Geomatics, University of Peshawar, Peshawar 25000, Pakistan; atta-ur-rehman@uop.edu.pk; 4 Faculty of Computer Science and Engineering, Macau University of Science and Technology, Macau 999078, China; xltian@must.edu.mo

**Keywords:** power tower damage, remote sensing, data augmentation, diffusion models, cross-resolution mismatch

## Abstract

**Highlights:**

**What are the main findings?**

- Modeling satellite-domain scale priors and applying frequency-domain pre-style adaptation to UAV-derived damaged references effectively alleviates scale inconsistency and spectral–textural discrepancy in cross-resolution diffusion-based inpainting.
- Feature statistical analysis combined with downstream change detection provides an effective framework for validating the quality and downstream usability of bi-temporal damage-generated samples.

**What are the implications of the main findings?**

- Diffusion-based inpainting models are highly sensitive to the input data distribution. Pre-adapting training samples in both scale and frequency domains is an effective way to mitigate scale-related artifacts and domain inconsistency in generated results.
- The proposed evaluation strategies support the practical use of synthetic imagery by enabling more reliable quality assessment prior to downstream applications.

**Abstract:**

Satellite-based assessment of power tower damage is essential for rapid disaster response but is challenged by the scarcity of damage samples and the cross-scale mismatch between close-range UAV imagery and satellite imagery. Existing data augmentation methods, including copy-based strategies and diffusion-based generation, often fail to produce reliable samples due to their dependence on the training data distribution and the lack of explicit control over object scale and domain discrepancy. To address these issues, we propose a scale-constrained and frequency-adaptive diffusion-based data construction framework that explicitly models the scale distribution prior of power towers in the remote sensing domain and incorporates frequency-domain adaptation before image generation. Specifically, scale-aware instance embedding is used to construct training samples that conform to satellite-scale statistics, while frequency-domain adaptation is introduced to reduce spectral and texture discrepancies between UAV-derived damaged references and satellite imagery. A diffusion-based inpainting model is then trained on the constructed dataset to reconstruct damage at original tower locations. The experimental results, including feature statistical analysis and downstream change detection validation, demonstrate that the proposed method achieves better alignment with real satellite-scale distributions, reduces geometric and spectral–textural inconsistencies, and improves boundary continuity and structural realism under cross-resolution conditions.

## 1. Introduction

Power towers are a critical component of modern power grids, widely distributed in forests and mountainous areas [1,2]. Due to their remote locations, post-disaster assessments face numerous challenges, especially during periods of increased risk from extreme weather events and large-scale natural disasters [3]. Earth observation satellites, with their wide coverage and high-frequency revisit capabilities [4], offer an efficient solution for rapid, large-scale post-disaster assessment of power tower damage.

However, in practice, obtaining samples of damaged power towers after disasters is subject to numerous limitations. Because power infrastructure is critical, minor damage is typically repaired quickly, resulting in limited opportunities for systematic data collection. While large-scale disasters cause widespread destruction, they occur relatively infrequently. Satellite revisit cycles, the availability of high-resolution sensors, and potentially adverse weather conditions during disasters further limit the acquisition of high-quality post-disaster satellite imagery. These factors impact downstream mission applications. In many cases, UAV imagery is more commonly used to document damage. For example, Bao et al. proposed BC-YOLO for detecting insulator defects from UAV inspection images [5], while Barreiro et al. investigated automated corrosion inspection of power transmission towers from close-range UAV images [6]. However, they mainly focus on component-level defects within the UAV image domain.

On the other hand, high-resolution satellite imagery of intact power towers is relatively easy to obtain. Open-access datasets such as the EPD dataset [7] and manually acquired satellite imagery from platforms like Google Earth provide sufficient examples of normal power towers. However, even with orthophotography, obvious differences remain between damaged UAV samples and satellite remote-sensing tower images, especially in target-scale representation. For hollow steel-frame targets like power towers, variations in spatial resolution and relative target size can lead to significant mismatches in structural distribution. Therefore, traditional data augmentation strategies in deep learning are insufficient to bridge these cross-scale differences if we want to apply damaged power tower UAV data for a satellite inspection training task.

To address this issue, we propose a scale-constrained and frequency-adaptive diffusion-based framework for synthetic data construction in satellite-based power tower damage assessment. The main contribution of this work lies in the application-oriented integration of scale distribution constraints, frequency-domain pre-style adaptation, and latent diffusion-based inpainting.

To summarize, our contributions are threefold:

(1) We propose a scale-constrained and frequency-adaptive diffusion-based synthetic data construction framework for satellite-based power tower damage assessment under cross-resolution mismatch. The framework is designed for a practical setting in which damaged tower references are mainly available from UAV imagery, while the target assessment task relies on satellite remote sensing images.
(2) We introduce two adaptation strategies before diffusion-based inpainting to improve the suitability of UAV-derived damaged references for satellite-domain reconstruction. Specifically, the satellite-domain scale prior reduces the apparent-scale mismatch between close-range UAV samples and satellite power tower scenes. And frequency-domain adaptation reduces spectral and texture discrepancies between UAV-derived damaged references and satellite backgrounds.
(3) We construct bi-temporal pre-event and post-event samples by repainting original tower regions in real satellite imagery and systematically evaluate their quality and downstream usability. The results of ablation and downstream change-detection experiments demonstrate that the proposed workflow reduces scale and texture inconsistencies and improves the usefulness of the generated samples for satellite-based damage assessment.

## 2. Related Work

### 2.1. Few-Shot Learning

Few-shot learning offers an approach that generalizes with only a small number of labeled samples. Existing methods primarily mitigate the impact of complex backgrounds and scale variations by improving representation robustness and class alignment.

One research direction is to enhance feature representation through architectural improvements, such as introducing Mamba-like attention mechanisms [8]. Alternatively, it explores generalization-enhancing training strategies or robust features to mitigate feature shifts in satellite imagery [9,10,11]. To stabilize class alignment under limited supervision, researchers have proposed label consistency classifiers and progressive regression mechanisms to improve metric learning [12].

Another research direction focuses on knowledge preservation. Since few-shot learning involves limited training data, introducing new classes for fine-tuning can lead to catastrophic knowledge loss; therefore, knowledge preservation mechanisms are needed [13]. Frameworks such as InfRes employ prototype calibration to support continuous adaptation [14]. Unsupervised approaches leverage unlabeled data to evolve representations for classification [15].

Besides architecture and training strategies, some researchers have attempted to incorporate prior knowledge to compensate for insufficient samples. For example, Ji et al. [16] attempted to augment the data by adding rotation variables or embedding category descriptions into features [17]. Recent research has sought to introduce Large Language Models (LLMs) as decision aids. For instance, Zheng et al. directly used LLMs for image classification [18]. Cheng et al. implemented a similar approach for power tower damage assessment, submitting detected suspected targets to an LLM to assist in decision-making [19].

Despite these advances, most few-shot frameworks implicitly assume that a limited support set is sufficient to capture the structural and scale variability in target categories. This assumption is problematic to hold in satellite-based power tower damage assessment tasks. Rare damage categories, feature mismatches across scales, and variations in imaging conditions can lead to severe shifts in distribution. In such cases, simply improving representation learning cannot address the lack of structural diversity. Introducing additional knowledge introduces new annotation and application costs or data security issues.

### 2.2. Constructing Synthetic Data

Since few-shot learning itself is challenging to apply to complex scenarios, constructing synthetic data has become a practical strategy to improve model robustness [20]. Existing data augmentation methods can be broadly categorized into traditional copy-based augmentation, generative adversarial network GAN-based generative modeling, and diffusion-based image synthesis.

Traditional data augmentation methods, such as geometric transformations and copy–paste operations, are widely used to increase the diversity of training samples. Ghiasi et al. demonstrated that transplanting the target to a new background improves segmentation training [21], while Wang et al. further applied it as pseudo-labels in semi-supervised learning [22]. These methods essentially preserve the original object’s features and lack natural transition edges.

To further enhance the realism of generated samples, GANs have been introduced to improve the naturalness of context fusion and the consistency of feature distribution. For example, combining the pix2pix model with copy–paste operations [23]. Ho et al. even combined it with disaster generation scenarios [24]. Kumar et al. considered using GANs for image inpainting [25]. However, overall, GANs generally lack smooth fusion capabilities, and scale issues are rarely studied [26].

In recent years, diffusion models have been increasingly widely used in sample data augmentation. Related research has introduced diffusion models into remote sensing fake sample generation tasks and incorporated conditional guidance mechanisms to construct visually consistent synthetic data [27,28]. Xu et al. used it to generate post-earthquake UAV photographic data [29].

Diffusion-based image inpainting has further expanded its application in regional simulations [30], increasing its usability and enabling masked reconstruction simulation experiments of disaster scenes. For example, Qu et al. and Ou et al. used region-specific masks to simulate post-disaster road damage, increasing the number of samples [31,32]. Immanuel et al. and Liu et al. further applied it to few-shot learning [33,34,35], enhancing contextual diversity. However, Immanuel et al. also recognized that although diffusion models can be applied to address data scarcity, applying them alone cannot directly address the scale problem caused by cross-resolution.

## 3. Materials and Methods

### 3.1. Data Sources

**Figure 1:**
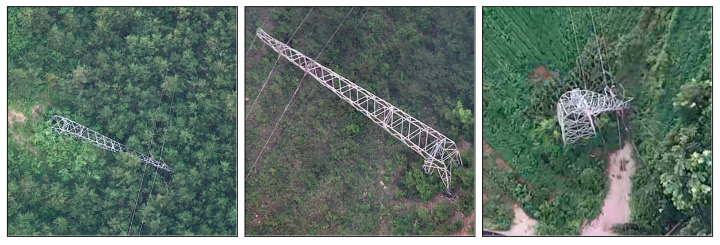
Examples of close-range UAV images captured in vegetated environments.

We used various datasets for this work, including the EPD tower dataset [7], the LoveDA dataset [36], our self-collected UAV-based power tower damage data shown in Figure 1, and satellite imagery data containing towers that we collected. We resized all data to 512 pixels.

#### 3.1.1. EPD Dataset

**Figure 2:**
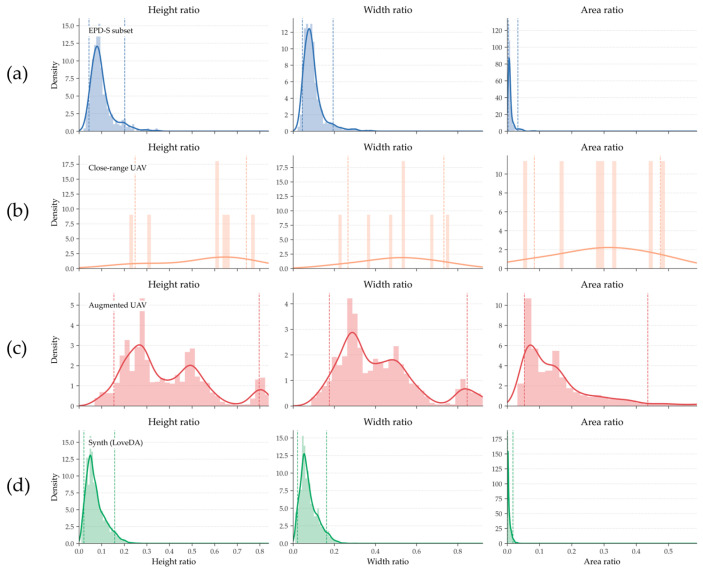
Statistical distribution of kernel density estimates for the height, width, and area ratio of bounding boxes of power towers (including damaged instances) under different datasets. The dashed lines represent the 5th to 95th percentile range, and the vertical axis represents the kernel density. Colored curves represent the kernel density estimates for different datasets, and the histograms represent the distributions of the corresponding ratios. (**a**) EPD-S subset. (**b**) Original close-range UAV imagery. (**c**) Enhanced UAV imagery. (**d**) Synthetic dataset generated from the original UAV imagery after distribution adjustment and processing with the LoveDA ensemble algorithm.

The EPD dataset was divided into two subsets by Qiao et al. We used the EPD-S subset. We primarily used this dataset to statistically analyze the distribution of power tower scales in the satellite domain. It contains optical satellite imagery with a spatial resolution of approximately 1m, containing over 3000 tower instances. The dataset contains various resolutions; so, it was first normalized to 512 px before computation, preserving as many towers as possible within the range. The proportion of tower dimensions in EPD is shown in Figure 2a.

#### 3.1.2. Close-Range UAV Damage Dataset

We obtained power tower damage samples from UAV inspection images acquired during on-site monitoring activities, with a resolution resized to 512 px. A total of 5 damaged tower images are available. In some images, the tower occupies a large portion of the frame. We collected all samples in environments with vegetation, as in Figure 1. Figure 2b shows their size distribution, and Figure 2c shows their direct data augmentation results.

#### 3.1.3. LoveDA Dataset

To supplement background semantic information during synthetic data construction, we used the LoveDA land cover dataset. The LoveDA dataset contains high-resolution satellite imagery with a 0.3 m spatial resolution, covering various land types. In the actual synthetic process, we randomly cropped a 512 × 512-pixel image patch from the Rural subset of LoveDA as the initial background to obtain richer, more natural landscape-oriented imagery.

#### 3.1.4. High-Resolution Background Dataset

We acquired those images from Google Earth imagery at a resolution of 0.3 m. We collected this data along power lines in Ya’an, Sichuan Province, China. The data was labeled in ArcGIS 3.6, with the label boxes slightly larger than the tower area to control the redrawing scope better. It was then automatically segmented into image tiles and downsampled to 512 pixels. Some image data may be stretched due to the mountainous terrain.

### 3.2. Core Method

#### 3.2.1. Method Structure

**Figure 3:**
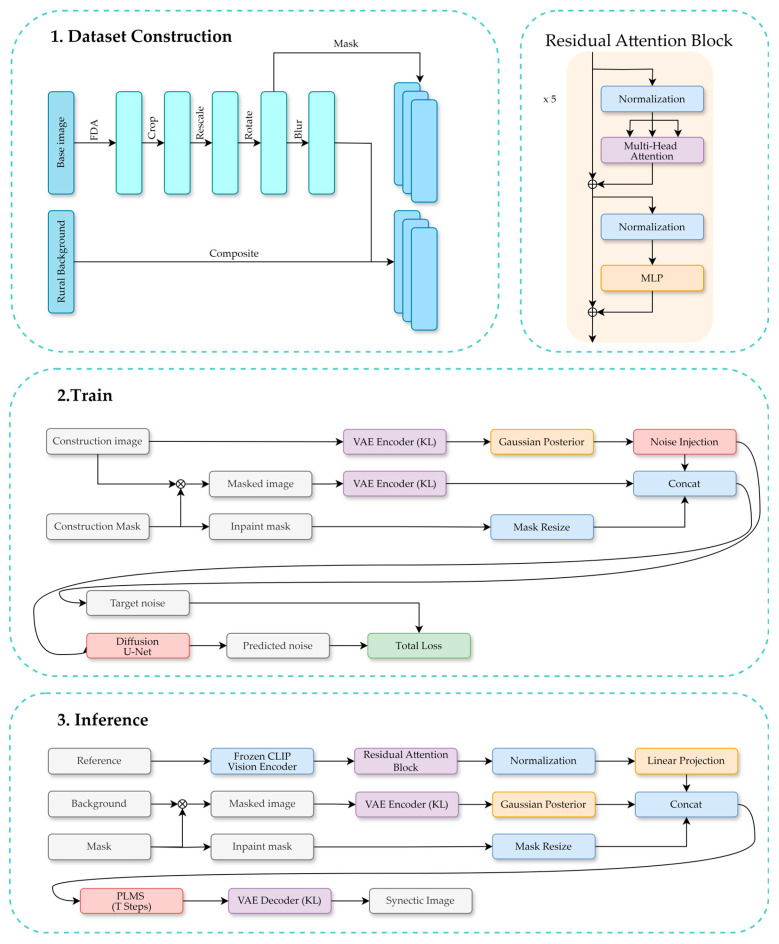
Overview of the proposed method. Scale-constrained synthetic damage samples are used to train a latent diffusion-based inpainting model. Satellite backgrounds with rural-style scenes are introduced to reflect the typical spatial context of power towers.

As shown in Figure 3, the main target of the proposed method is to generate realistic satellite imagery containing power tower damage using a limited set of UAV-captured close-range samples.

The main objective of this study is to construct damaged power tower samples that are consistent with the scale distribution of satellite imagery. However, this task cannot be achieved by directly training a standard diffusion model to generate complete remote sensing images. The available damaged tower samples are extremely limited and mainly collected from UAV imagery. However, the satellite images have much lower spatial resolution and different textural characteristics. Those differences make it difficult for a standard diffusion model to learn realistic damaged tower structures and satellite-like appearances simultaneously from close-range UAV images. In particular, directly using UAV-scale damaged samples may cause the model to learn inconsistent object proportions, unrealistic local textures, and unstable structural patterns in the generated satellite images.

The proposed framework first constructs scale-constrained damaged samples based on the empirical tower-size distribution observed in remote sensing images. This step reduces the geometric mismatch between UAV-derived damaged instances and satellite-domain tower objects, allowing the diffusion model to learn from training samples with more appropriate object scales and structural proportions. Domain adaptation is further introduced to narrow the appearance gap between UAV and satellite images by making the generated samples more similar to the satellite image style. These two preprocessing steps are not intended to generate final bi-temporal samples directly but to provide more suitable training data for the subsequent diffusion-based inpainting model.

After the scale and style adjustments, a latent-diffusion-based inpainting model is trained to reconstruct damaged regions of the tower. The use of latent diffusion reduces the difficulty of directly modeling dense pixel-level changes in high-resolution remote sensing images by representing the reconstruction process in a compressed latent space. Meanwhile, the inpainting formulation preserves the original satellite background and limits the generation process to the masked tower region. Therefore, the model does not need to generate the complete remote sensing scene from scratch. Instead, it just needs to reconstruct the locally damaged tower area under the guidance of scale-constrained and satellite-style reference samples.

#### 3.2.2. Prior Modeling of Scale Distribution

In high-resolution satellite image patches, power towers typically occupy a small proportion of the image area and can be regarded as small objects. In contrast, UAV close-range imagery captures significantly larger areas of tower damage, leading to substantial scale discrepancies and distinct texture characteristics. This scale inconsistency limits the direct applicability of UAV imagery to downstream detection tasks in satellite imagery.

Before training the diffusion model shown in Figure 3, we explicitly model a scale prior distribution to characterize the statistical properties of tower sizes in satellite imagery. We collect tower instances from the EPD power tower dataset. Since the EPD dataset mainly contains normal power towers, while our synthesis task focuses on damaged tower instances, the proposed prior is not intended to establish an exact distributional correspondence between normal and damaged towers. It provides an approximate target-size constraint estimated from real tower annotations.

For each annotated instance with bounding box dimensions (w, h) in an image of size (W,H), we define the normalized scale as follows:

(1) $$s_{w}=\frac{w}{W}$$

(2) $$s_{h}=\frac{h}{H}$$

where $\left(s_{w},s_{h}\right)$ represents the normalized bounding box dimensions extracted. To improve robustness, extreme samples outside a fixed percentile range are removed before estimating the prior parameters. Based on these processed empirical scale statistics, bounded marginal scale constraints are estimated and used as statistical constraints in the subsequent synthesis process.

Instead of directly sampling from the raw empirical distribution, which contains a small number of long-tailed large-scale samples, we adopt a bounded Gaussian scale prior to approximate the dominant satellite-domain scale range. Specifically, we estimate two bounded marginal scale priors for normalized width and height. The candidate marginal reference scales used to define the admissible target-size range are first sampled as follows:

(3) $${\tilde{s}}_{w}∼N\left(\mu_{w},\sigma_{w}^{2}\right)$$

(4) $${\tilde{s}}_{h}∼N\left(\mu_{h},\sigma_{h}^{2}\right)$$

where $\left(\mu_{w},\mu_{h}\right)$ and $\left(\sigma_{w},\sigma_{h}\right)$ denote the mean and standard deviation estimated from the processed empirical scale statistics. In implementation, the sampled values are clipped to the valid scale range estimated from real tower annotations so that extremely small or oversized synthetic instances are avoided. These marginal scale values are used to constrain the admissible target-size range rather than to independently deform the width and height of the damaged tower instance. During synthesis, the input damaged tower is resized using an aspect-ratio-preserving scaling operation:

(5) $$\left(w_{s},\;h_{s}\right)=\alpha \left(w_{0},\;h_{0}\right)$$

where $\left(w_{0},\;h_{0}\right)$ and $\left(w_{s},\;h_{s}\right)$ denote the original and resized bounding-box dimensions of the damaged tower instance, respectively, and α is the isotropic scaling factor.

Therefore, the scale distribution of synthesized samples $P_{s}\left(s\right)$ is softly guided by the prior $P_{r}\left(s\right)$ as follows:

(6) $$P_{s}\left(s\right)\approx P_{r}\left(s\right)$$

where the approximation indicates a soft statistical constraint on the dominant scale range rather than strict distribution-level matching.

#### 3.2.3. Scale-Constrained Dataset Construction

Simple augmentation approaches typically rely on geometric augmentation, such as rotation, scaling, and color perturbation. Though these operations introduce regional variations, they cannot alter the overall data distribution and effectively bridge the discrepancy between close-range UAV and satellite imagery.

**Figure 4:**
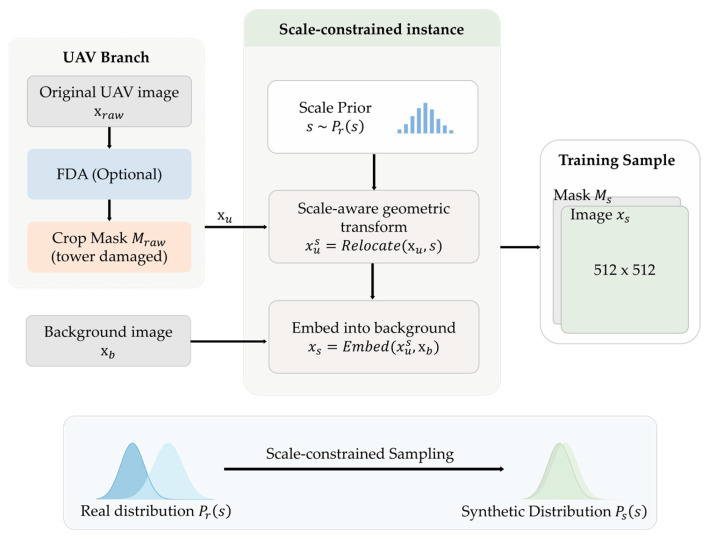
Scale-constrained dataset construction pipeline.

In contrast, the dataset construction process of the proposed method shown in Figure 4 is formulated from a distributional modeling perspective, explicitly controlling scale distributions, spectral characteristics, and spatial context. A key modification is to reconstruct the scale distribution of damaged instances, thereby alleviating the mismatch between close-range observations and high-resolution satellite imagery.

Specifically, the scale of each instance is modeled as a random variable following the empirical distribution derived in previous section. To ensure physical plausibility, the sampled scale is constrained within a predefined range:

(7) $$\bar{s}=clip\left(s,s_{min},s_{max}\right)$$

The instance is resized according to $\bar{s}$ while preserving its aspect ratio, enabling distribution-level consistency between synthetic samples and real satellite observations.

In addition to scale alignment, domain discrepancy between close-range UAV imagery and satellite imagery is reflected in low-frequency appearance statistics, such as color distribution and illumination patterns. Directly embedding close-range instances into satellite backgrounds often introduces visual inconsistencies and undermines the stability of diffusion-based reconstruction. To address this issue, a spectral alignment mechanism based on Fourier domain adaptation (FDA) is introduced [37,38].

Let $F\left(x\right)=A\left(x\right)e^{i\phi \left(x\right)}$ denote the Fourier decomposition of an image into amplitude A(x) and phase ϕ(x), where i is the imaginary unit. Given a UAV instance $X_{raw}$ and a satellite image $X_{t}$ adapted instance is constructed as follows:

(8) $$\hat{X}=F^{-1}\left(A\left(X_{t}\right)e^{i\phi \left(X_{raw}\right)}\right)$$

where the phase component preserves structural information, and the amplitude component transfers the global appearance statistics of the satellite domain. This formulation maintains the geometric integrity of damaged instances while improving domain consistency.

Geometric transformations are applied to the instances after scale and spectral alignment, within constrained ranges, to simulate variations in satellite imaging perspectives. After the transformations, the foreground region is localized from the cropped instance, and an axis-aligned minimum bounding rectangle is computed to construct a binary mask defining the initial reconstruction region. This mask provides a structured spatial constraint for the diffusion model and allows for subsequent morphological operations during data processing.

To further increase contextual diversity, background patches are sampled from the LoveDA dataset and used as composition canvases. The transformed instances are embedded into these backgrounds, followed by lightweight perturbations and blurring to refine boundary transitions.

#### 3.2.4. Damage-Conditional Diffusion Inpainting

**Figure 5:**
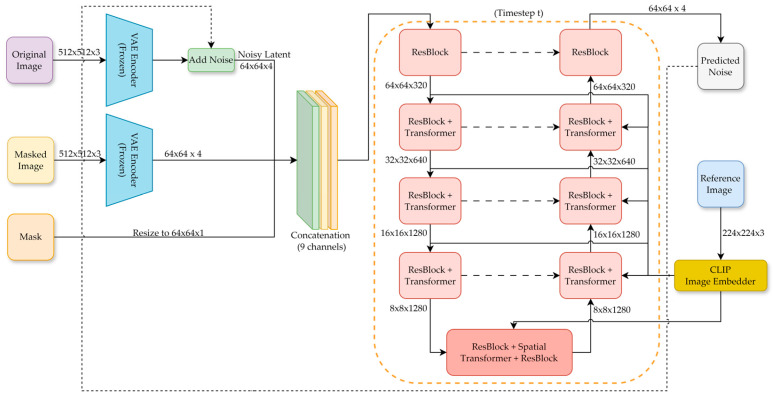
Detailed architecture and layer configuration of the latent diffusion-based inpainting model.

**Figure 6:**
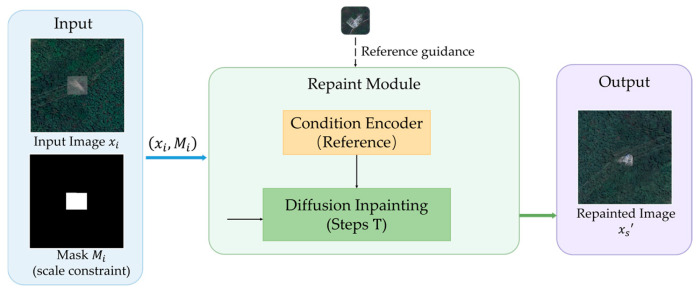
Synthetic construction for power tower damage.

After constructing synthetic satellite samples of damaged power towers with scale distributions similar to those observed in real remote images, we further introduce a diffusion-based inpainting model shown in Figure 5 to perform region repainting on real satellite images containing normal power towers, thereby constructing a bi-temporal damage dataset. Unlike most inpainting scenarios, the power tower damage reconstruction shown in Figure 6 requires replacing the original tower structure while preserving the background consistency within the interior.

The diffusion process is defined on latent variables [39], where the forward noising process perturbs clean data $x_{0}$ into a noisy state $x_{t}$:

(9) $$x_{t}=\sqrt{{\bar{\alpha }}_{t}}x_{0}+\sqrt{1-{\bar{\alpha }}_{t}}\epsilon ,\;\;\epsilon ∼N\left(0,I\right)$$

where ${\bar{\alpha }}_{t}$ denotes the cumulative noise schedule. In the reverse process, the model predicts the noise component conditioned on the masked input and reconstructs the target content within the masked region.

The conditional input is defined as follows:

(10) $$c=\left\{x_{\mathrm{masked}\;},m\right\}$$

(11) $$x_{\mathrm{masked}\;}=\left(1-m\right)⊙x_{0}$$

where m denotes the binary mask indicating the region to be repainted. Under this formulation, the model generates damaged structures inside the masked region while preserving the visible context.

The denoising network is optimized using a hybrid loss, written as follows:

(12) $$L_{\mathrm{total}\;}=L_{\mathrm{simple}\;}+\lambda L_{vlb}$$

with the standard noise prediction term:

(13) $$L_{\mathrm{simple}\;}=E_{x_{0},\epsilon ,t,c}\left[{\left\|\epsilon -\epsilon_{\theta }\left(x_{t},t,c\right)\right\|}_{2}^{2}\right]$$

where $\epsilon_{\theta }$ is the predicted noise and $L_{vlb}$ denotes the variational lower-bound term used to regularize the diffusion process.

During inference, classifier-free guidance maintains consistency between the generated content and the conditional input. The guided noise prediction is defined as follows:

(14) $${\hat{\epsilon }}_{\theta }\left(x_{t},t,c\right)=\epsilon_{\theta }\left(x_{t},t,\emptyset \right)+w\left(\epsilon_{\theta }\left(x_{t},t,c\right)-\epsilon_{\theta }\left(x_{t},t,\emptyset \right)\right)$$

where w is the guidance scale and ∅ denotes the unconditional input.

Finally, after the damaged content is generated inside the masked region, the repainted area is merged with the unchanged background according to the mask, yielding the final reconstructed image:

(15) $${x_{s}}^{\prime }=M_{i}⊙x_{d}+\left(1-M_{i}\right)⊙x_{i}$$

where $x_{d}$ denotes the generated result within the masked region and $x_{i}$ denotes the original satellite image. In this way, the tower region is replaced, while the surrounding background remains unchanged.

## 4. Results

### 4.1. Sample Feature Analysis

The diffusion model used for synthetic data generation was initialized from the SAMRS fine-tuned Stable Diffusion v1.5 weights [33,40]. Training was conducted on a single NVIDIA RTX Pro 6000 GPU (96 GB memory) (Nvidia Corporation, Santa Clara, CA, USA). The input resolution was set to 512 × 512 pixels, with a batch size of 24. The model was trained for 60–90 epochs until convergence. For environments with limited GPU memory (e.g., 32 GB), the batch size should be adjusted accordingly. The remaining hyperparameter settings are summarized in Table 1 and Table 2.

#### 4.1.1. Scale Distribution Consistency Evaluation

We first evaluate the prior statistical results of the composite distribution and the scale distribution of power towers in satellite imagery. Although the RS-Paint method provides a masking guidance scheme during the synthesis process [33], this constraint primarily controls the repaint location rather than the scale consistency of the spatial distribution of the generated results.

Although there are differences between damaged and standard towers, their actual bounding box scale distributions still overlap significantly due to differences in satellite imagery angles. The damage itself does not cause significant differences in scale.

Our comparison is based on 0.3 m resolution tower images from Google Earth imagery. The diffusion inpainting model tends to redraw the entire mask region; therefore, the tower mask region we use to guide image generation is slightly larger than the actual tower area to allow the model to redraw edge regions. To accelerate the statistical process, we apply the YOLO model [41] for semi-automatic annotation, followed by manual correction.

We assume the distribution domain of the real satellite tower scale is $P_{r\left(x\right)}$, and the distribution domain of the synthetic samples is $P_{s\left(x\right)}$, where x represents the normalized-scale ratio. To better reflect the performance differences between the synthetic samples, we use three primary metrics to measure the distribution differences.

The Wasserstein distance measures the minimum transportation cost between two distributions and is highly robust to changes in their shapes:

(16) $$WD\left(P_{r},P_{s}\right)=\underset{\gamma \in \Pi \left(P_{r},P_{s}\right)}{inf}{\left(E_{\left(x,y\right)∼\gamma }{d\left(x,y\right)}^{p}\right)}^{\frac{1}{p}}$$

For one-dimensional scale variables in this research (W-1), this study admits a simpler representation:

(17) $$W_{1}\left(P_{r},P_{s}\right)=\int \left|F_{r}\left(x\right)-F_{s}\left(x\right)\right|dx$$

where $F_{r}$ and $F_{s}$ represent the empirical cumulative distribution function (CDF).

The Kullback–Leibler (KL) divergence measures the difference between two probability distributions:

(18) $$D_{KL}\left(P_{r}\|P_{s}\right)=\sum_{x\in \chi }P_{r}(x)log\frac{P_{r}(x)}{P_{s}(x)}$$

Discrete probability estimation employs a uniform binning strategy applied to the support set of the scaling variable.

The Kolmogorov–Smirnov Statistic (KS) is used to measure the maximum local shift between two scale distributions is denoted as follows:

(19) $$KS\left(P_{r},P_{s}\right)=\underset{x}{sup}\left|F_{r}(x)-F_{s}(x)\right|$$

**Figure 7:**
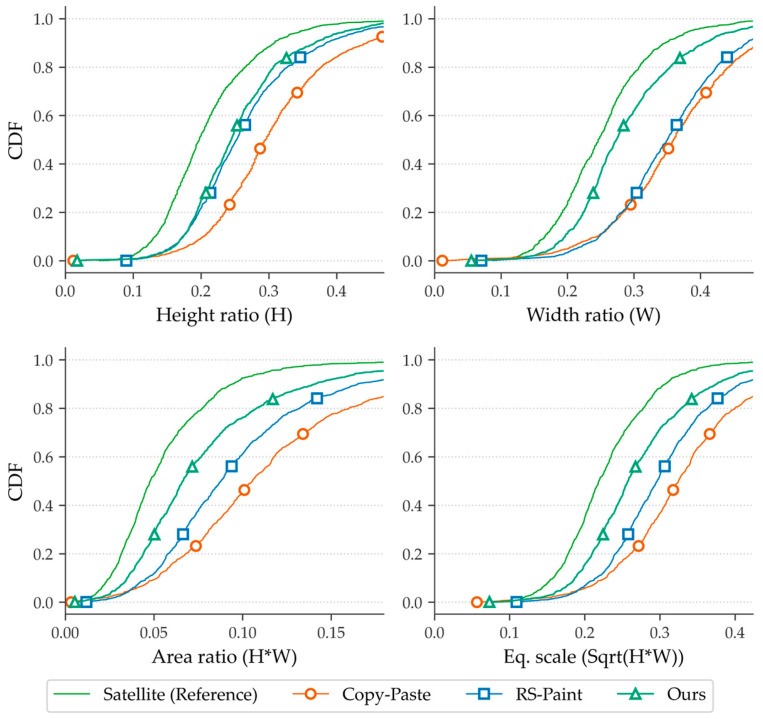
CDFs of bounding-box scale ratios under different synthesis settings. The distributions are compared with the real satellite data (SAT) as a reference. A closer alignment with the SAT distribution indicates better scale consistency and more effective distribution control.

We show results in Table 3 and the empirical cumulative distribution function (CDF) in Figure 7. The comparison focuses on the height (H), width (W), and area of the bounding boxes generated in the re-annotated YOLO format while using an equivalent scale bar as an auxiliary indicator for visualization.

As shown in Figure 7, the copy–paste method from Ghiasi et al. [21] exhibits a significant rightward shift across all indicators, indicating that the composite object is systematically enlarged. The RS-Repaint partially mitigates this bias. However, significant differences remain, particularly in the width direction.

In contrast, the statistics from the method we proposed are close to the CDF curves of real satellites for those dimensions. The bias consistently decreases across the entire range. This trend is similar to the results in Table 3, where we obtained the lowest *W*_1_, KL, and KS values across all indicators.

#### 4.1.2. Structural Consistency Evaluation

Beyond the requirement of scale alignment, we also test whether the generated results are structurally stable to avoid some issues, such as boundary gradient discontinuities, spectral distortions, or texture inconsistencies. These defects may look well in statistical data, but can significantly impact downstream learning tasks.

Those issues above are particularly critical for tasks like power tower damage assessment. The steel-framed towers in the images can sometimes blend into the background. Downstream studies may learn boundary patterns introduced during the synthesis process, thereby reducing generalization performance.

Boundary gradient continuity (BGC): To quantify boundary smoothness, we use a morphological adjustment mask to construct a bounding ring around each synthesized object. $G_{\mathrm{in}}$ and $G_{\mathrm{out}}$ represent the gradient magnitude changes in the inner and outer boundary zones, respectively. It is defined as follows:

(20) $$BGC=\left|\mu \left(G_{\mathrm{in}}\right)-\mu \left(G_{\mathrm{out}}\right)\right|\;$$

where μ(⋅) represents the mean operator, and the smaller the value, the smoother the transition of the object boundary.

Spectral Wasserstein-1 distance (Spec-W1): The stability of the global structure is then evaluated by comparing the amplitude spectra obtained from the Fourier transform, i.e., by assessing the frequency domain. $A_{r}$ and $A_{s}$ represent the spectral amplitude distribution within the ring of the original satellite image and the composite image. Their similarity is measured by the first-order Wasserstein distance.

(21) $$W_{1}\left(A_{r},A_{s}\right)=\int \left|F_{A_{r}}\left(a\right)-F_{A_{s}}\left(a\right)\right|da$$

Texture distribution consistency (TDC): finally, it is evaluated by the chi-square divergence between the texture histograms of the normalized rings:

(22) $$TDC\left(P_{r},P_{s}\right)=\sum_{x}\frac{{\left(P_{r}(x)-P_{q}(x)\right)}^{2}}{P_{r}(x)+\epsilon }$$

ϵ is a small constant used for numerical stability. A lower value indicates greater consistency in local texture patterns.

Table 4 shows the structural consistency statistics for two annular band-width settings, with the tower distribution in authentic satellite imagery as a reference. In the BGC index, our method’s mean is significantly lower than the baseline method under both annular band widths. It is significantly closer to the statistical range of the original satellite imagery. Overall, our method more closely approximates the original distribution and does not significantly alter the redraw results, demonstrating good consistency with the structure. For example, when the ring width is set to 50 + 5 pixels, it achieves 1.99 ± 2.11, which is very close to the original distribution’s 1.33 ± 1.20. In the Spec-W1 index and TDC index, our method also remains stable without significantly compromising consistency.

#### 4.1.3. Generative Reliability Evaluation

While scale and structural consistency metrics have demonstrated the effectiveness of the diffusion inpainting model in controlling scale and boundaries, they do not guarantee that the generated samples strictly adhere to our expectations. In actual test scenarios, insufficient training may lead to two types of failures: (1) semantic deviation of the generated results from the reference image, resulting in uncontrollable generation; (2) incorrect adjustment of background areas other than the trunk. To further evaluate the reliability of our generation method, we conducted a semi-quantitative manual evaluation for these two potential problems.

First, we evaluated the consistency of damage type, denoted as Guidance Acc. From the perspective of the reference image, we evaluated whether the synthesized tower structure conformed to the expected damage type. If the trunk structure was close to the expected damage type, we considered the sample valid.

Second, we evaluated whether the background was preserved, denoted as Background Acc. Since the original damaged image had a background dominated by green vegetation, the model might incorrectly learn that there should be green space around the damaged tower during redrawing. If no apparent semantic inconsistencies were observed (e.g., vegetation was generated on bare land), the sample was considered acceptable.

The semi-quantitative evaluation was conducted by three evaluators with relevant domain expertise, including one researcher in power systems, one senior remote sensing image interpretation engineer, and one expert in remote sensing image annotation. Each evaluator independently inspected the generated samples according to the criteria described above. For each image, the final pass/fail judgement was determined by majority voting; that is, a sample was considered valid only when at least two evaluators judged that it met the minimum criterion. In addition, inter-observer consistency was assessed using the agreement ratio, which measures the proportion of samples for which all three evaluators gave the same judgement, either all valid or all invalid.

**Figure 8:**
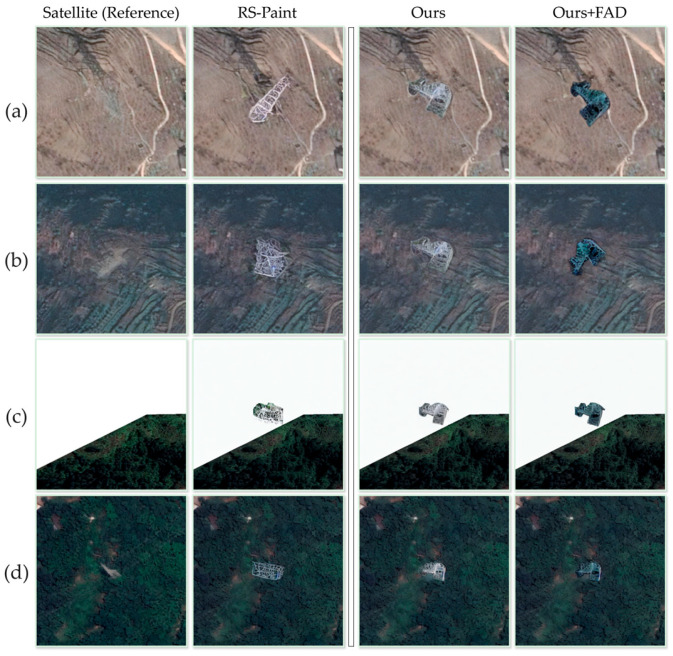
Examples of sample generation by the model are shown in four sets of images: (**a**) on bare land, (**b**) near hilly farmland, (**c**) an incorrect blank label, and (**d**) near a forest.

Table 5 summarizes the expert-based semi-quantitative evaluation results, while Figure 8 further illustrates this. The original diffusion-based remediation framework exhibits severe instability in structure-guided control, with a guidance consistency of only 9.53%. The high guidance agreement ratio for RS-Paint also suggests that evaluators consistently identified its guidance failures rather than producing highly subjective judgements. In contrast, our method achieves 99.3% relative accuracy, indicating that we effectively prevent semantic bias. Although the baseline shows relatively high background consistency at 92.62%, it almost always fails when the repainted scene lacks vegetation, which suggests an implicit background bias. Our method achieves 99.72% background consistency and demonstrates greater robustness under different land cover conditions.

In addition, the FDA was also used explicitly for satellite-style transfer; since this was applied to the training set, the changes are mainly reflected in the redrawn damaged towers.

### 4.2. Downstream Validation Based on Change Detection

Based on the three groups of samples generated in the previous sections, we constructed three bi-temporal change detection datasets and trained the same M-CD model [42]. M-CD was adopted as the downstream validation model because it follows the widely used Siamese bi-temporal change detection paradigm, which uses pre-change and post-change remote sensing images to predict a binary change mask. This input–output form is consistent with our validation task. In addition, using a fixed representative model avoids introducing extra model-specific effects from comparing multiple change detection architectures or adding highly task-specific branch modifications. Since the purpose of this experiment was not to benchmark change detection architectures but to evaluate the downstream learnability of different synthetic datasets, all datasets were evaluated using the same M-CD architecture, initialization, training settings, dataset proportions, and evaluation protocol.

**Figure 9:**
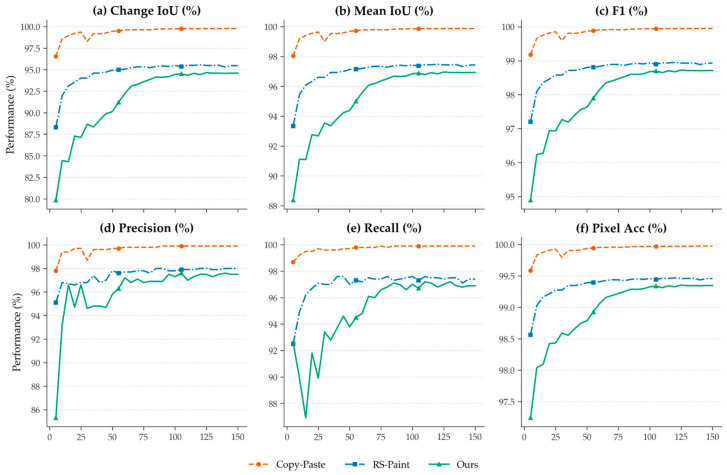
Training curves of the same change detection network using datasets generated by different sample construction methods.

As shown in Table 6 and Figure 9, all three datasets exhibit a similar overall trend: the validation performance gradually improves with increasing training epochs and eventually stabilizes. The copy–paste baseline achieves the fastest convergence and the highest change detection scores. However, this result should not be directly interpreted as evidence that copy–paste produces more realistic synthetic samples. The copy–paste operation introduces sharp, deterministic discontinuities between the pasted damaged region and the surrounding satellite background, making the changed regions easier for the change detection model to segment.

In contrast, RS-Paint and the proposed method show slower convergence and lower final IoU values. It indicates that the generated changes are less trivially separable from the unchanged background. In particular, the proposed method starts with lower validation performance and converges more slowly, achieving stable performance later in training. This phenomenon is consistent with the objective of the proposed framework, which aims to generate damage regions that are constrained by satellite-domain scale statistics and better integrated with the surrounding background, rather than simply producing changes that are easy to segment.

Since the damaged tower regions occupy only a small portion of the image, the IoU of the changed class is more informative for analyzing the learning difficulty of the generated samples:

(23) $$IoU=\frac{\left|P\cap G\right|}{\left|P\cup G\right|}$$

where P denotes the predicted change region and G represents the ground truth region. The background class usually obtains high IoU for all methods; therefore, the variation in mIoU is less sensitive than the change-region IoU. For this reason, the following analysis focuses mainly on the IoU of the changed class.

To further interpret the above change detection results, we analyzed the feature-level relationship between changed and unchanged regions in the constructed bi-temporal samples. For each bi-temporal sample, feature vectors were extracted from the changed regions (masked regions) and unchanged background regions.

Fisher’s separability was used to measure the discriminability between the changed-region features and the background-region features:

(24) $$J=\frac{{\left\|\mu_{c}-\mu_{b}\right\|}_{2}^{2}}{\sigma_{c}^{2}+\sigma_{b}^{2}+\epsilon }$$

where $\mu_{c}$ and $\mu_{b}$ are the mean feature vectors of changed-region features and background-region features. $\sigma_{c}^{2}$ and $\sigma_{b}^{2}$ denote the corresponding intra-region feature variances, and ϵ is a small constant used to avoid division by zero.

We further calculated cosine similarity to evaluate the feature consistency of the bi-temporal samples:

(25) $$S\left(f_{A},f_{B}\right)=\frac{f_{A}\cdot f_{B}}{{\left\|f_{A}\right\|}_{2}{\left\|f_{B}\right\|}_{2}+\epsilon }$$

where $f_{A}$ and $f_{B}$ denote the feature vectors extracted from the pre-change and post-change images at the same spatial location, respectively. The binary change mask was used to determine whether each location belonged to the changed region or the unchanged background region. The change cosine $S_{c}$ was obtained by averaging the cosine similarity over the changed regions, while the background cosine $S_{b}$ was obtained by averaging the cosine similarity over the unchanged background regions. The cosine gap was then defined as follows:

(26) $$G=S_{b}-S_{c}$$

A larger cosine gap indicates a stronger feature difference between the changed regions and the unchanged background, which means that the synthetic changes are easier for the change detection model to distinguish. Conversely, a smaller cosine gap suggests weaker artificial separation between the generated damage and the surrounding satellite background.

As shown in Table 7, the copy–paste method obtains the highest Fisher’s separability and the largest cosine gap, indicating that its changed regions are highly distinguishable from the unchanged background. This explains why copy–paste achieves the highest mIoU in the downstream change detection experiment. However, such high separability also suggests stronger artificial discontinuities between the pasted damaged region and the surrounding satellite background. In contrast, the proposed method achieves the lowest Fisher’s separability and cosine gap while maintaining the highest change-region cosine similarity. It indicates that the damaged regions generated by the proposed method maintain stronger feature consistency within the changed regions. The background cosine similarity also shows that the proposed method reduces background disturbance compared with RS-Paint, although copy–paste still obtains the highest background cosine because it directly preserves most unchanged background pixels.

These analytical metrics for bi-temporal images indicate that the proposed method achieves a better balance between change-region feature consistency and background preservation. In contrast, change detection networks can easily learn highly separable change patterns, such as those introduced by copy–paste operations. However, such obvious discontinuities rarely appear in real satellite images. Therefore, higher change-detection accuracy does not necessarily mean better sample realism, and the lower mIoU of the proposed method should be understood as a result of more natural integration between the generated damage and the surrounding background.

### 4.3. Ablation Study

To distinguish the contributions of the scale prior and FDA, we conducted an ablation study using two groups of metrics. The scale-related metrics evaluate whether the synthesized damaged tower instances follow the empirical satellite-domain size distribution, while BGC and Spec-W1 evaluate the local background consistency around the synthesized region.

As shown in Table 8, scale prior consistently reduces the W-1 distance from satellite instances, especially with FDA, and effectively constrains the synthesized damaged instances to a more reasonable satellite-domain target-size range.

The image structural consistency metrics further show the contribution of the FDA adaptation. Compared with the baseline and the variant without, the test with the results from the full framework achieves lower values and is similar to satellite imagery. It suggests that the FDA helps reduce local appearance discrepancy, while scale prior also works for it.

In conclusion, it shows that the scale prior and the FDA contribute to different but complementary aspects of the synthesis process. The scale prior mainly improves target-scale consistency, while the FDA mainly improves local appearance adaptation. The full framework provides a more balanced result across scale distribution and background consistency, supporting the combination of scale-constrained sample construction and domain adaptation in the proposed cross-resolution synthesis framework.

### 4.4. Validity and Sensitivity Analysis of the Scale Prior

To further analyze the validity of the proposed scale, we conducted a statistical analysis using real tower annotations from the EPD dataset. Since the EPD dataset primarily contains normal tower instances, these statistics are not used to establish a precise distributional correspondence with damaged towers. Instead, they provide an approximate reference for constraining the target size of synthesized damaged tower samples.

It should be noted that all image patches in this study are generally processed into 512 × 512 pixels before subsequent synthesis and evaluation. Therefore, the scale prior is designed from a normalized-scale perspective. As shown in Table 9, under the original EPD spatial resolution of 1 m/pixel, the mean box height and width of tower instances are 50 px and 52 px, respectively, with corresponding standard deviations of 27 px and 33 px. After removing 10% of the extreme samples, the valid height and width ranges are 25–107 px and 24–129 px. These statistics are used to construct the bounded marginal-scale prior to constrain the synthesis scale of UAV-derived damaged-tower samples within satellite-image patches. In practice, the scale parameters should be adjusted based on the image size and spatial resolution of the target imagery.

We further analyzed the relationship between normalized width and height. As shown in Table 10, the Pearson’s and Spearman’s correlation coefficients are 0.5963 and 0.4015, respectively, indicating moderate geometric coupling between the two dimensions. This result is consistent with the actual implementation of the proposed method, in which the width and height of damaged tower instances are not treated as fully independent variables. Instead, their relative proportion should be preserved during resizing. Therefore, the bounded marginal priors are used to constrain the admissible target-size range, while the final resizing operation preserves the aspect ratio of the input damaged tower instance.

To evaluate the sensitivity of the proposed scale prior, we set the estimated mean to ±10% and the standard deviation to ±20%. For each, we used the KL divergence to compare them. As shown in Table 11, with moderate perturbations, the height mean ranges from 0.0949 to 0.1102, while the width mean ranges from 0.1004 to 0.1168. These results indicate that the proposed target-size constraint does not change abruptly under moderate parameter variations.

## 5. Discussion

### 5.1. Impact of Scale Priors

The experimental results clearly demonstrate the significant role of incorporating prior satellite-scale distribution data from the EPD and LoveDA datasets as the initial synthetic training dataset. As shown in Figure 6, the model trained with our method outperforms the original method in terms of width when synthesizing tower damage within the same mask area, more closely approximating the actual distribution. Table 3 further illustrates this point. The KL divergence obtained by the RS-Paint method even exceeds that of the copy–paste method in height, indicating a significant deviation.

**Figure 10:**
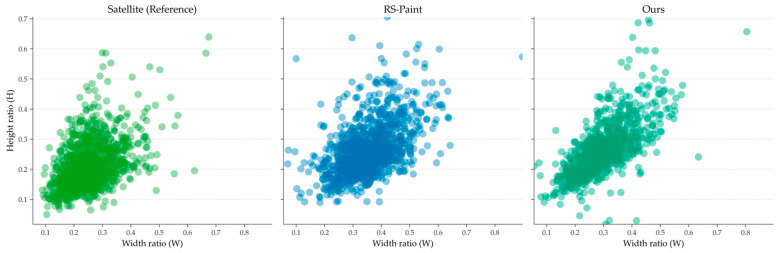
Size distribution of objects generated by real satellite samples (**left**), baseline method (**middle**), and our method (**right**).

Figure 10 compares the joint scale feature space of samples generated by our method, satellite data, and baseline methods. The real samples exhibit a concentrated and positively correlated distribution in both width and height. The baseline methods, on the other hand, show a wider, right-shifted, and more dispersed distribution. In contrast, the joint scale distribution generated by our method is closer to the true data distribution.

The essence of image inpainting diffusion models lies in optimizing local visual plausibility, prompting the rendering of background and object content within the masked region to conform to the transition. However, it cannot explicitly constrain changes in foreground geometry relative to the global image scale. This limitation becomes more pronounced as spatial resolution differences increase in cases of cross-scale mismatch. UAV imagery, especially low-altitude UAV imagery, typically captures objects at a larger relative scale, providing richer information on object and background texture structure. On the other hand, features in satellite remote sensing images are smaller, with more distinct texture features and reduced complexity. Therefore, training a diffusion model directly on UAV imagery may lead to the incorrect assumption that the objects should be repainted larger. While adjusting the mask size can directly constrain the generation range, texture structure issues may still exist.

This comparison explains why models trained directly on UAV samples always appear larger. Without explicit scale-constrained prior knowledge, diffusion models tend to overdraw within the mask area, filling it with content that appears reasonable. This finding should apply not only to the synthesis of samples from UAV to satellite remote sensing images but also to situations requiring rendering across all scales. The model renders oversized objects because it cannot understand the relationship between the scale and spatial resolution of the redrawn area. Another possible solution is to embed a scale-aware module within the model and train it on labeled multi-scale remote sensing data, but this would incur high, unnecessary costs for small-scale applications.

### 5.2. Change Detection and Sample Generation

**Figure 11:**
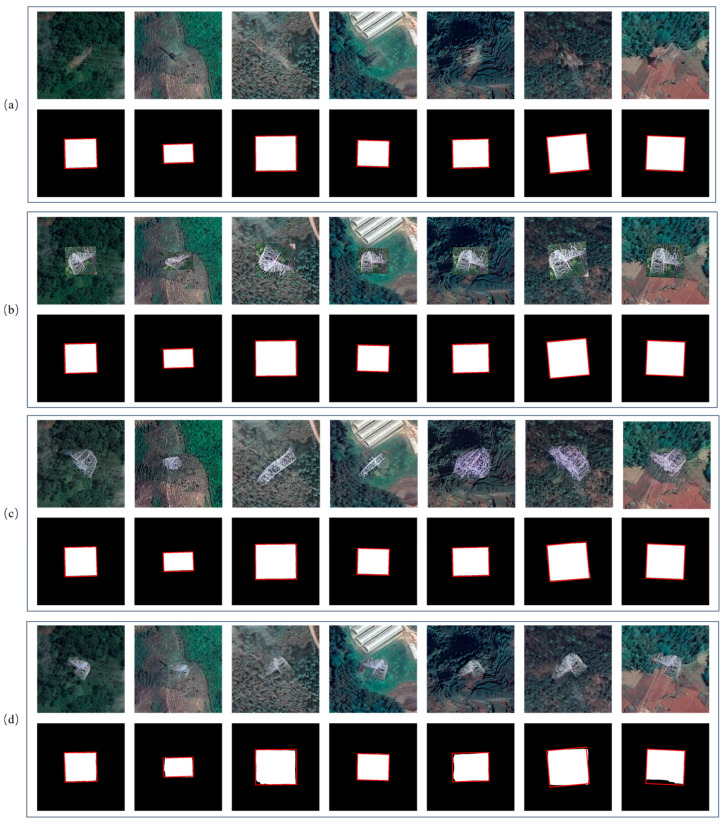
Qualitative test results of the change detection model on datasets constructed by different generation methods. (**a**) Original image and ground truth annotations. (**b**) Copy–paste method and corresponding predictions. (**c**) RS-Paint method and corresponding predictions. (**d**) Proposed method and corresponding predictions.

Figure 11 presents qualitative examples of the change detection results on datasets constructed by different generation methods. To supplement this visual comparison, the quantitative change-detection results are reported in Table 6, and the feature-level separability analysis of changed and unchanged regions is reported in Table 7.

Change detection is sensitive to appearance differences between bi-temporal images and can therefore reflect whether the generated changes are trivially separable from the background. As shown in Figure 11, different sample construction methods lead to different prediction behaviors. Figure 11a shows the original satellite image and ground truth annotation. Figure 11b–d shows the generated post-change samples and the corresponding predictions obtained from the copy–paste method, RS-Paint, and the proposed method, respectively.

In the copy–paste results, the predicted change regions are close to the annotated regions because the pasted damaged areas introduce sharp and easily distinguishable boundaries. The RS-Paint results show more shape variation, but the generated regions still largely follow the annotated areas. In contrast, the proposed method produces more noticeable boundary and shape variations, indicating that the generated damage regions are less dependent on rigid copy-based geometry and are more integrated with the surrounding satellite background.

This observation is consistent with the quantitative results in Table 6 and Table 7. Although the copy–paste method achieves the highest mIoU, Table 5 shows that it also has the highest Fisher’s separability and cosine gap, indicating that its changed regions are more easily distinguished from the background. The proposed method achieves the lowest separability and cosine gap while maintaining the highest change-region cosine similarity. These results suggest that the proposed samples are less trivially separable and provide more background-consistent synthetic changes, even though this makes the downstream change detection task more difficult.

### 5.3. Practical Considerations and Limitations

This study was conducted under extremely limited real-damage conditions. Only five damaged UAV images were available for constructing damage references, which is insufficient for directly training a robust detection or change-detection model. Therefore, the data synthesis process had to be carefully designed to account for the available sample condition.

For this task, full-scene image generation is not suitable because it requires the model to learn both the background distribution and the damaged tower structure from very limited samples. It may easily lead to biased generation, unstable outputs, or overfitting to repeated visual patterns. In contrast, diffusion-based inpainting provides a more practical solution because it only reconstructs the masked region while preserving the surrounding satellite background. It allows existing high-resolution satellite images of normal power towers to be used as background inputs and enables the construction of bi-temporal change-detection samples.

However, the inpainting model still requires sufficient, properly distributed training data. In our preliminary experiments, directly fine-tuning the diffusion inpainting model using the initial UAV-derived samples failed to produce reliable results. The combined effects of limited sample diversity, domain discrepancy between UAV and satellite imagery, and background bias in the original samples mainly caused it. Simple geometric or mathematical augmentation increased the number of samples, but it could not fully eliminate the scale mismatch and background contamination problem. Table 5 and Figure 8 further corroborate this point. The diffusion model tends to generate vegetation near the damaged tower, regardless of the actual background. Figure 8c shows this with a blank background, indicating that the model did not correctly interpret the tower object from the training set background. This indicates that this model internally incorrectly correlates vegetation with tower damage, demonstrating the significant impact of dataset bias on the generation behavior. Despite using images of the same type of damage for guidance, the reliability of the generation is relatively low. Incorrect scale information makes it difficult to depict the tower damage structure accurately.

Therefore, scale-constrained instance construction and FDA-based domain adaptation were introduced before fine-tuning the inpainting model. The scale constraint aligns the damaged tower instances more closely with the target satellite-domain size distribution. At the same time, the FDA reduces the discrepancy in appearance between UAV-derived damage references and satellite backgrounds. Together with the use of diverse satellite backgrounds, these steps reduce the risk of overfitting to the limited damaged UAV samples and improve the robustness of the generated results under different background conditions.

In addition, to reduce the manual cost of selecting and initializing raw images for synthetic sample construction, candidate tower regions can be obtained using object detection models, general segmentation models such as SAM [43], or existing vector data of power tower locations. These approaches can help screen remote sensing images, localize normal power towers, and initialize inpainting regions, thereby reducing the workload required for manual inspection and sample preparation.

Nevertheless, the current framework still has several limitations. First, the generation quality is constrained by the initialization and construction of synthetic damage samples. Although the constructed samples improve the diversity of backgrounds and target scales, real power tower damage may appear in many different forms in disaster scenarios. Due to the limited number of available damage references, downstream models may still encounter damage patterns that are not covered by the synthetic training set in real emergency response scenarios. Therefore, for practical deployment, synthetic data construction should be combined with other few-shot learning methods so that downstream models can adapt to potential new damage patterns.

Second, the quality of generated samples still depends strongly on data preparation. Power towers often feature thin components, hollow structures, fine internal gaps, and frequent vegetation, which make accurate foreground–background separation of damaged tower references difficult. It may introduce edge artefacts during synthetic sample construction. A possible improvement is to optimize the input samples before diffusion-based inpainting, for example, by using more accurate mask refinement, boundary smoothing, alpha blending, or edge-aware background reconstruction to improve the transition between the damaged tower instance and the surrounding background.

Third, shadow generation remains an important issue for future work. The damaged UAV references used in this study, in fact, lack sufficient shadow information. In real scenes, shadows may vary with shooting angle, tower damage patterns, and so on. Without this physical context, diffusion-based inpainting may generate locally plausible textures, but it cannot guarantee physically consistent shadow patterns. Future work may introduce additional physical information to improve the controllability and realism of generated shadows.

## 6. Conclusions

This paper proposes a scale-constrained data augmentation framework to address the cross-scale problem in generating satellite samples from close-range UAV-damaged images. This method introduces a scale distribution based on statistical data to construct training samples for the diffusion model that conforms to scale priors. It combines scale-aware instance embedding with diffusion-based image inpainting to ensure structural consistency and scale appropriateness of the generated samples. Furthermore, FDA is introduced to mitigate domain differences between UAV and satellite imagery, thereby improving visual consistency and background compatibility.

Graph feature analysis and downstream change-detection experiments demonstrate that the proposed method aligns better with the true satellite-scale distribution than existing methods. The generated samples exhibit smaller geometric deviations, better boundary continuity, and more realistic structural patterns across resolution conditions. These results indicate that explicitly constraining the scale distribution and introducing domain adaptation are crucial for generating reliable data. Moreover, the results show that diffusion-based data augmentation methods are highly sensitive to the distribution of training data, and alignment at the distribution level plays a key role in improving generation quality.

Despite these improvements, some limitations remain. The extremely limited number of real-world damage samples restricts the diversity of generated structures and may still lead to inconsistencies in complex scenarios. Future work will focus on introducing stronger structural or physical constraints to improve generation reliability further and on exploring more robust domain-adaptive strategies. Furthermore, it will investigate how to extend the proposed framework to other remote sensing objects and tasks to evaluate its generalizability.

## Figures and Tables

**Table 1:** Hyperparameter used on model training phase.

Hyperparameter	Value	Hyperparameter	Value
Base learning rate	1.0 × 10^−5^	Learning-rate scheduler	Lambda Linear Scheduler
Maximum epochs	100	Batch size	24
Scale factor	0.18215	Diffusion timesteps	1000
Arbitrary mask ratio	0.3	Unconditional conditioning ratio	0.2
Bounding-box ratio	0.05~0.2	Input image size	512

**Table 2:** Hyperparameters used on the model inference stage.

Hyperparameter	Value	Hyperparameter	Value
Sampler	PLMS	Scale	5
Sampling steps	20	Background Size	512
Background Size	512	Reference Image Size	224

**Table 3:** Scale statistical distribution differences. Each row of indices represents the difference between the scale distribution index of power towers and that in the satellite sample. The smaller the value, the smaller the difference.

Method	W-1			KL			KS		
	H	W	AREA	H	W	AREA	H	W	AREA
Copy–Paste	0.10	0.11	0.06	0.87	0.88	0.96	0.51	0.54	0.55
RS-Paint	0.06	0.10	0.04	0.57	0.92	0.78	0.32	0.52	0.46
Ours	0.05	0.04	0.02	0.51	0.23	0.25	0.29	0.19	0.27

**Table 4:** Structural consistency assessment result. Statistical results are differentiated using two sampling intervals: 25 px and 55 px. The first value in the table cell is the statistical mean, and the second is the standard deviation.

Method	BGC		Spec-W1		TDC	
	20 + 5 px	50 + 5 px	20 + 5 px	50 + 5 px	20 + 5 px	50 + 5 px
SAT	1.44 ± 1.35	1.33 ± 1.20	0.31 ± 0.02	0.11 ± 0.02	0.02 ± 0.01	0.02 ± 0.01
Copy–Paste	21.83 ± 9.94	19.21 ± 8.93	0.33 ± 0.03	0.13 ± 0.03	0.06 ± 0.02	0.05 ± 0.02
RS-Paint	4.10 ± 3.69	3.54 ± 3.11	0.32 ± 0.02	0.12 ± 0.02	0.02 ± 0.01	0.02 ± 0.01
Ours	2.28 ± 2.58	1.99 ± 2.11	0.31 ± 0.02	0.11 ± 0.02	0.02 ± 0.01	0.02 ± 0.01

**Table 5:** The results of the reliability checks generated on the dataset are not included, as the copy–paste method is meaningless. The “accuracy” here refers only to the consistency of pass/fail under manual inspection, not the accuracy of predicted classification.

Method	Guidance Acc (%)	Background Acc (%)	Count	Guidance Agreement Ratio (%)	Background Agreement Ratio (%)
RS-Paint	9.52	92.62	1071	94.49	98.23
Ours	99.35	99.72	1071	98.04	99.44

**Table 6:** Downstream change detection performance of bi-temporal datasets constructed by different methods.

Method	Epoch	IoU (%)	Rec (%)	F1 (%)	mIoU (%)
Copy–Paste	40	99.179	99.6	99.807	99.5354
	150	99.783	99.9	99.949	99.877
RS-Paint	40	94.606	97.6	98.715	96.934
	150	95.478	97.4	98.929	97.433
Ours	40	88.346	92.8	97.193	93.362
	150	94.600	96.9	98.713	96.932

**Table 7:** Feature separability and cosine similarity analysis of changed and background regions in the bi-temporal samples. Bold values indicate the metrics where our method achieves superior performance.

Method	Fisher’s Separability	Change Cosine	Background Cosine	Cosine Gap
Copy–Paste	1.9233	0.8786	0.9997	0.1211
RS-Paint	1.0549	0.8808	0.9872	0.1064
Ours	**0.9348**	**0.8963**	0.9941	**0.0978**

**Table 8:** Ablation results for scale distribution and background consistency. All metrics were computed using the same evaluation protocol described in the previous sections. Lower values indicate better consistency. SAT denotes the reference statistics calculated from real satellite image patches and is not an ablation variant. Bold values indicate the metrics where our method achieves superior performance.

Method	Scale Prior	FDA	Scale W-1			BGC		Spec-W1	
			H	W	AREA	20 + 5 px	50 + 5 px	20 + 5 px	50 + 5 px
SAT	-	-	-	-	-	1.44 ± 1.35	1.33 ± 1.20	0.31 ± 0.02	0.11 ± 0.02
Baseline	×	×	0.056	0.101	0.042	4.10 ± 3.69	3.54 ± 3.11	0.32 ± 0.02	0.12 ± 0.02
+FDA	×	✓	0.054	0.062	0.030	4.03 ± 6.23	3.45 ± 5.19	0.32 ± 0.02	0.12 ± 0.02
+Scale	✓	×	0.049	0.054	0.029	2.50 ± 2.39	2.21 ± 1.97	0.31 ± 0.02	0.11 ± 0.02
+FDA + Scale	✓	✓	**0.046**	**0.036**	**0.024**	**2.28 ± 2.58**	**1.99 ± 2.11**	**0.31 ± 0.02**	**0.11 ± 0.02**

**Table 9:** Scale statistics of power tower instances based on the EPD dataset.

Variable	Mean (px)	Std (px)	Valid Range (px)	Mean Ratio
Height	50	27	25–107	0.0977
Width	52	33	24–129	0.1016

**Table 10:** Correlation analysis between normalized width and height.

Metric	Correlation Coefficient	*p*-Value
Pearson’s correlation	0.5963	2.37 × 10^−197^
Spearman’s correlation	0.4015	3.80 × 10^−80^

**Table 11:** Sensitivity analysis of the scale prior assumption.

Setting	Height Mean	Height KL	Width Mean	Width KL
Baseline	0.1023	0.2231	0.1088	0.3385
μ × 0.9	0.0949	0.2130	0.1004	0.3275
μ × 1.1	0.1102	0.2562	0.1168	0.3616
σ × 0.8	0.1002	0.1562	0.1052	0.2444
σ × 1.2	0.1047	0.3147	0.1117	0.4359

## Data Availability

Publicly available datasets were used in this study, including EPD and LoveDA. Additional imagery from Google Earth was utilized for analysis. The UAV-collected damage samples and the generated datasets are not publicly available due to data usage restrictions, but are available from the corresponding author upon reasonable request.

## Abbreviations

The following abbreviations are used in this manuscript:

UAV	Unmanned Aerial Vehicle
GAN	Generative Adversarial Network
CLIP	Contrastive Language-Image Pre-training
PLMS	Pseudo Linear Multistep Method
FDA	Fourier Domain Adaptation
VAE	Variational Autoencoder
W-1	Wasserstein-1 distance
KL	Kullback–Leibler divergence
KS	Kolmogorov–Smirnov Statistic
CDF	Cumulative Distribution Function
BGC	Boundary Gradient Continuity
Spec-W1	Spectral Wasserstein-1 distance
TDC	Texture Distribution Consistency
IoU	Intersection over Union
mIoU	Mean Intersection over Union
